# A 7-Year Survey (2015–2021) in One Pediatric Hospital (Brasov, Romania) on Rotavirus Gastroenteritis Specified as Community- or Hospital-Acquired Infection in Young Children

**DOI:** 10.3390/tropicalmed8120509

**Published:** 2023-11-27

**Authors:** Ioana Arbanas, Vlad Monescu, Niculina Dragomir, Larisa Diana Sauciuc, Emanuela Cojocaru, Katalin Csutak, Bianca Elena Popovici, Pandaru Andreea, Spirea Elena-Daniela, Raluca-Ileana Lixandru, Laura Bleotu, Oana Falup-Pecurariu

**Affiliations:** 1 Children’s Clinical Hospital Brasov, 500063 Brasov, Romania; ioana.arbanas@yahoo.com (I.A.); dragomirniculina@gmail.com (N.D.); larisa_dia@yahoo.com (L.D.S.); cojocaru_ema@yahoo.com (E.C.); csutak.katalin68@gmail.com (K.C.); biancadr@yahoo.com (B.E.P.); andreeapandaru@yahoo.com (P.A.); despirea@gmail.com (S.E.-D.); ralucalixandru@gmail.com (R.-I.L.); laurableotu@yahoo.com (L.B.); oanafp@yahoo.co.uk (O.F.-P.); 2 Faculty of Medicine Brasov, Transilvania University, 500019 Brasov, Romania; 3 Faculty of Mathematics, Transilvania University, 500091 Brasov, Romania

**Keywords:** rotavirus gastroenteritis, hospital acquired gastroenteritis, community acquired gastroenteritis, Romania, rotavirus

## Abstract

This project is an observational, descriptive study evaluating frequencies of rotavirus disease in hospitalized children aged less than 5 years old between 2015 and 2021 in the Pediatric Hospital of Brasov, Central Romania. The study compares socio-demographic (age, sex, place of living and ethnicity), clinical, and treatment aspects between community-acquired rotavirus gastroenteritis (CARG) and hospital-acquired rotavirus gastroenteritis (HARG). During that period, 1913 hospitalized children had a rapid positive immunichromatographic rotavirus test from stool specimens. Among them, 1620 (84.6%) were CARG and 293 (15.4%) were HARG. CARG conditions represented 28.5% of all acute hospitalized gastroenteritis (*n* = 5673) whereas HARG represented 5.2%. Around the same percentage of urban children were seen in CARG as in HARG (58.5% (*n* = 948) for CARG and 56.3% (*n* = 164) for HARG). About 64.9% (*n* = 1052) of CARG cases were from Roma population, and 66.5% (*n* = 195) in HARG. The age group with the highest frequency of the disease was 12 to 24 months old for both CARG and HARG. The average hospital duration was 5.09 days for CARG and 7.62 days for HARG. Diarrhea was the principal symptom in both CARG and HARG (92.6% (*n* = 1500) for CARG and 93.9% (*n* = 275) for HARG). Most CARG patients (61% (*n* = 989)) were treated for symptomatic management with iv fluids. Most HARG (60.4% (*n* = 177)) were treated for symptomatic management with iv fluids and antibiotics. A significant seasonal shift to a later period in the year was observed during the last registration year of 2021, possibly due to the COVID-19 epidemic. The seasonal disease burden of rotavirus infection in children remains high in hospital care in Romania, which may justify the systematic introduction of rotavirus vaccination across the whole country.

## 1. Introduction

Rotavirus disease has a high morbidity in children under the age of 5 years and reached almost 40% of hospital admissions for diarrhea [1]. However, the burden may vary by country in Europe [2]. Rotavirus has a high rate of infectivity and high resistance in environmental conditions being stable at low temperature (4–20 Celsius degrees) and low humidity, and is even more stable in stools [3]. These characteristics may play a critical role in the dissemination process of the virus, which can lead to hospital-acquired rotavirus gastroenteritis (HARG) if hygienic prevention and control measures are not strictly applied in those institutions. In the United States before 2004 (pre-vaccine era), there were around 16,000 to 18,000 hospitalizations each year with HARG [4]. In Europe, rotavirus gastroenteritis leads to between 75,000 and 150,000 hospitalizations and up to 600,000 medical visits annually [5]. In Western Europe, HARG is responsible for 47–69% of all hospital-acquired gastroenteritis [6]. A few studies on rotavirus gastroenteritis enrolling large groups of children in Romania were published in the English literature, dealing with the epidemiological and clinical aspects of community-acquired rotavirus gastroenteritis (CARG) and HARG [7,8]. In the study “Hospital-based surveillance to estimate the burden of rotavirus gastroenteritis in children below five years of age in Romania”, Anca IA et al. show that the clinical pattern of rotavirus gastroenteritis was more severe compared to non-rotavirus gastroenteritis, with higher hospitalization rates and higher HARG incidence rates [8]. What has not been studied so far is the potential frequency difference in rotavirus disease in specific subgroups in Romania, such as children coming from urban versus rural areas, and factors such as being member of the Roma population or not. The latter represents about 8% of the population [9]. This population was one of the targets of this study.

The present study brings therefore an overview of the current situation of rotavirus gastroenteritis in one specific location in Romania, which is the Brasov area of Central Romania. It evaluates annual frequencies, clinical, epidemiological and treatment management characteristics between CARG and HARG. This study wants to demonstrate the level of rotavirus disease burden in Central Romania that could be greatly alleviated through vaccination when applied as a routine mass immunization program, which has not been implemented in the country at present.

## 2. Materials and Methods

### 2.1. Study Population

The study population included all children aged 0–60 months hospitalized between January 2015 and December 2021 with acute diarrhea at the Children’s Clinical Hospital of Brasov, for which a systematic testing of their stool showed a positive test result for rotavirus. Brasov county has around 546,600 inhabitants, with an estimated number of 32,000 (5.85%) children aged under 5 years of age. The Children’s Clinical Hospital of Brasov is the only pediatric hospital in the area and referral hospital for all nearby counties. The Children’s Clinical Hospital of Brașov has an average of 220 beds available with an average admission rate of 9055 cases per year during the observation period. Between 2015 and 2022 an average of 7628 children aged 0–60 months were hospitalized.

In 2022 the European Council estimated that approximately 1.85 million Roma live in Romania, which is 8.32% of the Romanian population [9]. They represent one of the largest communities of the Roma population in a European country. Because of this large presence, it was investigated whether rotavirus disease could have a different frequency of occurrence, thus leading to different rates of hospitalizations, amongst Roma children.

### 2.2. Methodology

This project is observational, but it first had a retrospective period (first 2 years) followed by a prospective study design since 2017 where the criteria of assessment remained the same in each period. Systematic testing of the stool samples occurred during each period when the main symptom for being admitted to the hospital was diarrhea. The children reported by the microbiology laboratory of the hospital with a positive stool specimen for rotavirus were considered for this evaluation. Meanwhile, all children during the observation who were hospitalized for other reasons than diarrhea at entrance but were experiencing diarrhea during their stay were also systematically tested for rotavirus at diarrhea onset. Those with a positive test result were also included in this evaluation.

#### 2.2.1. Lab Testing

The hospital laboratory used a rapid diagnosis test using the immunochromatography method. The tests used for the detection of rotavirus in stool samples were:

- Between 2015 and 2017, the CerTest Rotavirus + Adenovirus one step combo card test with a sensitivity > 99% and a specificity > 98% according to the manufacturer (Biotec S.L, Zaragoza, Spain) [10];
- Between 2018 and 2019, the Healgen Rotavirus and Adenovirus Combo Rapid Detection Kit with a sensitivity > 96.8% and a specificity > 97.7% (Healgen Scientific LLC, Houston, TX, USA) [11];
- for the years 2020–2021, the Rotavirus and Adenovirus Combo Rapid Test Cassette from Tody Laboratories with a relative sensitivity of 97.3% and a relative specificity of 97.1% (Tody Laboratories Int., Bucharest, Romania) [12].

The immunochromatography method was also used for Adenovirus antigen detection, Norovirus, *Clostridium difficile* (GDH antigen, and A and B toxins), and *Campylobacter* spp.

For *Adenovirus*, we used the following: between 2015 and 2017: CerTest Rotavirus + Adenovirus one step combo card test (Biotec S.L, Zaragoza, Spain) [10]; between 2018 and 2019: Healgen Rotavirus and Adenovirus Combo Rapid Detection Kit (Healgen Scientific LLC, Houston, TX, USA; and between 2020 and 2021: Rotavirus and Adenovirus Combo Rapid Test Cassette (Tody Laboratories Int., Bucharest, Romania).

The *Norovirus* antigen detection was performed starting January 2020 with the CerTest Norovirus card test (Biotec S.L, Zaragoza, Spain).

For *Clostridium difficile* (GDH antigen, and A and B toxins), we used the following tests: between 2015 and 2019 CerTest One step Clostridium difficile antigen GDH, Toxin A and Toxin B Combo card test (Biotec S.L, Zaragoza, Spain); and between 2020 and 2021: Clostridium Difficile GDH + Toxin A + B Rapid Test Cassette (Hangzhou Biotest Biotech Co., Ltd., Hangzhou, China).

For *Campylobacter* spp., the CerTest Campylobacter one step card test (Biotec S.L, Zaragoza, Spain) between 2015 and 2019 and the NADAL^®^ Campylobacter Test (Nal Von Minden GmbH, Moers, Germany) between 2020 and 2021 was used in the absence of an incubation thermostat at 42 degrees Celsius.

Fecal specimens were processed by standard microbiology methods to isolate *Salmonella* spp., *E. coli enteropathogenic* and *Klebsiella* spp. in newborns. The samples were inoculated on MacConkey, Hectoen. Cefsulodin–Irgasan–Novobiocin Agar was used for the isolation of *Yersinia enterocolitica*. Some of the samples have also been inoculated on Selenite F Broth medium for the selective enrichment of *Salmonella* spp.

#### 2.2.2. Other Information

Information on the socio-demographic aspects, clinical manifestations of the disease, and the treatment aspects were collected from electronic medical records and patient files from the hospital archive.

### 2.3. Case Definition

Acute diarrhea: three or more loose stools in 24 h.

Community-acquired rotavirus gastroenteritis (CARG): a child hospitalized for acute diarrhea with a positive stool rapid antigen test for rotavirus at admission.

Hospital-acquired rotavirus gastroenteritis (HARG): acute diarrhea with a stool rapid antigen test positive for rotavirus in a child hospitalized for a reason other than diarrhea with/without vomiting, occurring at least 48 h from admission. The rapid stool tests were performed at symptoms onset. If a child was hospitalized for other causes than diarrhea and vomiting 7–10 days prior to the present hospitalization, the detected rotavirus gastroenteritis episode was then also considered as HARG [13].

### 2.4. Data Management and Analysis

The frequencies of CARG and HARG were calculated as proportions using the number of CARG and HARG as the numerator and the total of acute diarrhea cases hospitalized in The Children’s Clinical Hospital of Brasov over the 7 years study time as the denominator. Microsoft Excel 365 package was used to generate descriptive summary statistics (mean, median, range, and standard deviation (SD)) together with Matlab Statistics and Machine Learning Toolbox for significance testing of the proportions. The description includes the number of rotavirus gastroenteritis per month per year (the seasonality of the disease), the sociodemographic parameters (the area of living, ethnic group, and sex), the age groups, the duration of hospitalization, the associated comorbidities, the clinical manifestation of rotavirus gastroenteritis (the average number of loose stools per day, the average number of episodes of vomiting per day, the degree of fever, and the presence of dehydration), the associated stool infections, and the treatment management options.

### 2.5. Ethics Statement

The study was approved by the Ethical Committee of the Clinical Children’s Hospital Brasov (17149/27 August 2019). All ethical considerations regarding personal data were strictly respected.

## 3. Results

### 3.1. Socio-Demographic Aspects

A total of 1913 children, aged 0 to 60 months old, with positive stool test results for rotavirus gastroenteritis (RG) were observed during the period of 2015 to 2021. Overall, 1620 (84.6%) were CARG, and 293 (15.4%) were HARG. The total number of acute diarrhea cases hospitalized between 2015 and 2021 was 5673 cases. The average proportion of RG over the 7-year study period was 33.7% compared to all acute diarrhea events. For CARG, that proportion was 28.6% (range: 21.6–39.1%), and for HARG, it was 5.2% (range: 2.5–8.9%).

Table 1 reports significant differences in the CARG population between urban and rural origin, a much higher presence of the disease in Roma children, and more in girls than boys. This was also the case for HARG, except for gender, with more boys suffering than girls.

**Figure 1:**
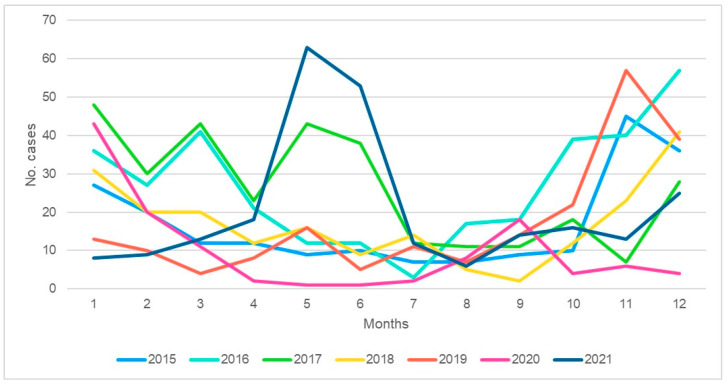
Distribution of community-acquired rotavirus gastroenteritis (CARG) cases according to month and year.

**Figure 2:**
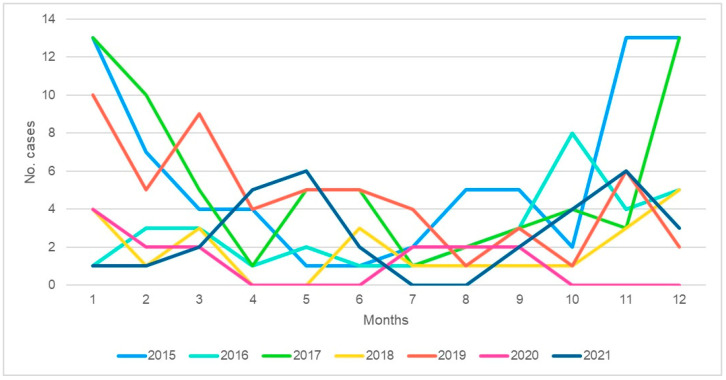
Distribution of hospital-acquired rotavirus gastroenteritis (HARG) cases according to month and year.

The highest percentage of cases for CARG was in the year 2016, and for HARG, it was in the year 2015. The monthly distribution of the cases per year is presented in Figure 1 and Figure 2. The months with the high frequencies are from October until April in the subsequent year. There were two peaks in May for CARG observed in 2017 and 2021. The last one is specific as the peak happens as a silo manifestation for 2 months only, with few cases outside that period.

**Figure 3:**
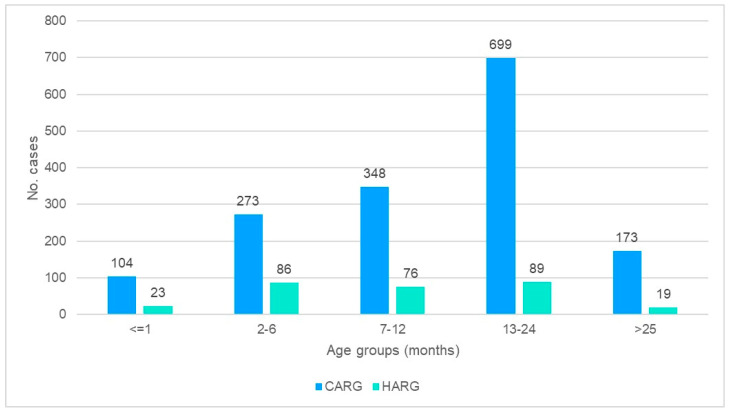
Age distribution of study patients. Comparison between community-acquired rotavirus gastroenteritis (CARG) and hospital-acquired rotavirus gastroenteritis (HARG).

The average age for CARG was 15.5 months (range: 10 days–60 months), and for HARG, it was 12.3 months (range, 15 days–60 months). The highest percentage of CARG cases was recorded in the age group of 13–24 months old (43.1% (*n* = 699)). For the HARG cases, there are two predominant age groups: 13–24 months (30.3% (*n* = 89)) and 2–6 months (29.3% (*n* = 86)). Overall, 10.6% (*n* = 173) of CARG patients were older than 25 months, and 6.4% (*n* = 19) for HARG patients (Figure 3).

The mean duration of hospitalization was 5.09 days ± 3.37 SD (median 4.0, range = 1–27 days) for CARG patients and 7.62 days ± 5.09 SD (median 6.0, range = 1–42 days) for HARG patients. The total number of hospitalization days for CARG was 7799 days, and 2219 days for HARG. The average number of days from symptoms onset until presentation to the emergency room was 2.37 ± 2.08 SD (median: 2; range = 0–21 days) for CARG and 1.84 days ± 1.98 SD (median 1; range = 0–7 days) for HARG. Out of all HARG (293 cases), 118 cases (40.3% of HARG) had a previous hospitalization 7–10 days prior the last one. The three most common primary reasons for hospitalization for HARG were acute pneumonia (46% (*n* = 135)), bronchiolitis (20.4% *n* = 60)), and urinary tract infections (6.14% (*n* = 18)).

Out of all children, only one, belonging to the CARG group, was completely vaccinated against rotavirus disease according to the child’s vaccination booklet.

### 3.2. Clinical Manifestations of Rotavirus Gastroenteritis

During hospitalization, 63.5% (*n* = 1216) of the children had fever above 39 degrees Celsius (64.1% for CARG and 60.4% for HARG). Dehydration was found in 98.9% of the children (99.3% (*n* = 1610) in CARG and 95.5% (*n* = 280) in HARG). The main symptoms of RG are presented in Table 2.

A total of 9.5% (*n* = 182) of the positive stool samples for RG were co-infected with another pathogen. In total, 9.07% (*n* = 147) of CARG were associated with co-infection and 11.9% (*n* = 35) for HARG. Next Table 3 reports the co-infection agents identified in CARG and HARG. The three most common co-infections in CARG were Adenovirus, *Campylobacter*, and *E. coli enteropathogenic*. For HARG, the three most common co-pathogens were Adenovirus, *E. coli enteropathogenic*, and Verotoxin (VT1 and 2).

In total, 8.58% (*n* = 139) of CARG patients had one additional stool pathogen, and 0.49% of CARG patients (*n* = 8) had two or more stool pathogens identified beside rotavirus. Overall, 10.9% (*n* = 32) of HARG patients had one additional stool pathogen, and 1.02% of (*n* = 3) HARG patients had two or more additional stool pathogens besides rotavirus.

Out of all these associated stool pathogens, 88 children presented at least one bacterial enteropathogen (23 children in HARG group (7.84%) presented at least one bacterial enteropathogen and 86 children in CARG group (5.30%)).

### 3.3. Treatment Management

For CARG, 989 cases (61%) were treated using symptomatic management and intravenous (iv) fluids. For HARG, 177 cases (60.4%) were treated using symptomatic management, iv fluids, and antibiotic (Table 4).

Symptomatic management included probiotics, calcium carbonate, diosmectite, trimebutinum, simethicone, and metoclopramide. Out of all 806 children receiving antibiotics, 247 children (30.6%) continued the antibiotic treatment that was started at home due to associated bacterial infection (236 children in CARG and 11 children in HARG). The rest of the children were put on antibiotics due to the associated bacterial infection (111 children in CARG and 91 children in HARG) or because of severe dehydration, poor clinical status, or as part of the neonatal fever management protocol (247 children in CARG group and 110 children in HARG).

## 4. Discussion

Over the years, few studies have been published in the English literature about rotavirus gastroenteritis in children in Romania. They showed that HARG comprised 22.4% of the cases and was more frequent in children between 3 to 6 months old [7]. Another study showed that the incidence of HARG in the central part of Romania was 0.52 per 1000 days of hospitalization, which is in the range reported for the whole of Europe [8,14]. In this study of the central part of Romania, the proportion for CARG being admitted in hospital care over the 7-year observation period was 28.5% of all acute diarrhea cases, with the highest number of 39.1% in 2016, and for HARG, the proportion was 5.16%, with the highest number of 8.99% in 2015.

Our study made a few interesting observations related to the subgroup identification with higher rates of RG, the high use of antibiotics amongst RG cases, the difference in RG spread across age groups when experiencing CARG or HARG, and the recent seasonality shift of the RG infection spread.

The first observation was in the subgroup of Roma children, with a % of rotavirus disease that was significantly higher in both CARG and HARG conditions compared to non-Roma children (see Table 1). If the spread of Roma people is homogeneous across the country, there should be no difference to be observed in the frequency of disease between Roma and non-Roma, and the expected number of CARG for Roma should have been around 49 cases. The observation is 20 times higher. The same is seen for HARG. There is a much higher concentration of the disease in this ethnic group. Reasons that can explain that situation could be the poor health condition of the Roma population in general [15] linked to a poor education and income level [16]. Their health condition compared to the non-Roma population includes a higher prevalence of chronic health diseases [17], which leads to a shorter life expectancy [18]. Moreover, fewer members of the Roma population are enrolled in social health insurance systems [19]. A study in Serbia showed that the Roma pediatric population presented statistically more comorbidities, more malnutrition, the presence of anemia, and may have a longer length of hospital stay with a higher number of laboratory tests and drugs recommended compared with non-Roma children [20]. An improvement in their social conditions should enhance better outcomes related to the rotavirus disease situation. They should most benefit of an appropriate vaccination program.

CDC stated that severe cases occur in children mostly between the age of 3 months to 3 years old [21]. Our study indicated that the age group most affected in both CARG and HARG patients was 13 to 24 months old. It was however seen that in the HARG group, the 2-to-6-months-old age group also had a high number of events, equivalent to the 13–24 age group. This may indicate that the hospital spread of the virus is much different from the community-acquired infection. The high number of infants who have contracted HARG can be due to the hospital admission rate of very young children with severe morbidity (mostly respiratory), by which they are more susceptible to other infections, with longer hospital stay as a consequence (see the Section 3).

We found a moderate number of co-infections in the rotavirus-positive stool samples with viruses and bacteria in CARG and HARG patients, with a slightly higher percentage in HARG patients (11.9% versus 9.07% in CARG). The most common co-infection was Adenovirus for both groups. The presence of co-associated stool pathogen brings up the question of which pathogen is the primary cause of the gastroenteritis. This could be linked to the seasonal spread of the disease, for which rotavirus infection is very specific in that respect from October to April.

The use of antibiotic treatment was high in our study in HARG patients. Out of all children receiving antibiotics, 30.6% continued their antibiotic treatment that was initiated at home due to the associated bacterial infection. The rest of the children were given antibiotics due to observed bacterial co-infection, severe dehydration, poor clinical state and young age. One study showed that in low- and middle-income countries, 12.6% of the cases of gastroenteritis received unnecessary antibiotics [22]. A more recent study showed that the only effective treatment for rotavirus gastroenteritis are synbiotics (pro-biotics and pre-biotics) and zinc [23]. However, probably one of the most important aspects regarding antibiotic treatment is the rise of its resistance, which today is one of the most significant public health threats [24]. According to the European Public Health Alliance, Romania is moving into a dangerous situation of being in the “red zone” of the excessive use of antibiotics and the rise of several bacteria that are drug-resistant [25].

What is interesting is that the appearance of the peak of the disease is now shifting to a later and more intense period in the year, as noted in the last observation year of 2021. It could be caused by the post-pandemic spread of COVID-19 virus, with the application of specific measures to attenuate its spread. This was also observed with other infections in children, such as RSV, flu infection, and pneumonia, and needs a close follow-up to determine what will happen in the coming years.

An important limitation of the study is the lack of identification of rotavirus serotypes due to the absence of a PCR analysis method during the study period. Meanwhile, a study between 2008 and 2016 showed a worldwide relative decline in rotavirus gastroenteritis by 39.6% in countries that have implemented rotavirus vaccination [26]. There is evidence that the vaccine against rotavirus gastroenteritis is highly effective as it can prevent up to 95% of severe rotavirus gastroenteritis that need hospitalization in infants [5]. In Romania, rotavirus vaccine is not included in the National Immunization Program (NIP), but the vaccine is available on the private market. It is estimated that vaccine-use against rotavirus gastroenteritis in Romania today is below the 8%. In 2014, Preda A.D et al. estimated that the implementation of rotavirus vaccination in the NIP could avoid around 82,581 mild cases, 511,328 moderate cases associated with physician visits, and 3075 severe RG cases associated with hospitalizations. This may result in a 2.4 million EUR direct and indirect cost offset, estimated through the reduction in hospital stays and the reduction in the productivity loss of caregivers [27]. We believe that important steps towards vaccination are required to improve the current situation of better rotavirus disease control in Romania.

## Figures and Tables

**Table 1:** Number of cases and percentage of the sociodemographic parameters studied (urban and rural areas, Roma population, and gender) and comparison between community-acquired rotavirus gastroenteritis (CARG) and hospital-acquired rotavirus gastroenteritis (HARG).

	CARG			HARG				
Socio Demographic Parameters	Nbr. Cases	%	Chi-Square Test	Nbr. Cases	%	Chi-Square Test	Total Nbr	Total %
Urban	948	16.7%	*p* < 0.01	164	2.9%	*p* < 0.01		
Rural	672	11.8%		129	2.27%			
Roma	1052	18.5%	*p* < 0.01	195	3.43%	*p* < 0.01		
Non-Roma	568	10%		98	1.74%			
Female	858	15.1%	*p* < 0.01	139	2.45%	*p* < 0.01		
Male	762	13.4%		154	2.73%			
Total RG	1620	28.6%		293	5.2%		1913	33.7%
All diarrhea							5673	

**Table 2:** Main symptoms (diarrhea and vomiting) of community-acquired rotavirus gastroenteritis (CARG) compared to hospital-acquired rotavirus gastroenteritis (HARG).

	CARG (*n* = 1620)					
	Nbr. Cases	%	Average Stools/Day	SD	Median	Range/Day
Diarrhea	1500	92.60%	4.94	2.37	5	0–20
Vomiting	1267	78.20%	3.89	2.83	3	0–20
	**HARG (*n* = 293)**					
Diarrhea	275	93.90%	5.06	2.07	5	0–20
Vomiting	187	63.80%	2.88	2.55	3	0–15

**Table 3:** Associated stool pathogens in children with community-acquired rotavirus gastroenteritis (CARG) and hospital-acquired rotavirus gastroenteritis (HARG).

	CARG (*n* = 1620)		HARG (*n* = 293)	
Stool Pathogen	Nbr. Cases	%	Nbr. Cases	%
*Adenovirus*	75	4.62%	16	5.46%
*Campylobacter*	31	1.91%	4	1.36%
*E. coli entheropathogenic*	28	1.72%	9	3.07%
VT1, VT2	21	1.29%	5	1.70%
*Klebsiella* spp.	2	0.12%	1	0.34%
*Salmonella* spp.	2	0.12%	2	0.68%
*Clostridium difficile*	1	0.06%	1	0.34%
*Yersinia* spp.	1	0.06%	0	0
*Norovirus*	1	0.06%	0	0

**Table 4:** Management treatment in children with community-acquired rotavirus gastroenteritis (CARG) and hospital-acquired rotavirus gastroenteritis (HARG).

	CARG (*n* = 1620)		HARG (*n* = 293)	
Treatment	Nbr. Cases	%	Nbr. Cases	%
Symptomatic management + IV fluids	989	61%	116	39.5%
Symptomatic management + IV fluids + ABX	629	38.8%	177	60.4%
Oral fluids + monitoring	2	0.12%	0	0%
Total	1620		293	

## Data Availability

Data are contained within the article.
